# OCT4 Potentiates Radio-Resistance and Migration Activity of Rectal Cancer Cells by Improving Epithelial-Mesenchymal Transition in a ZEB1 Dependent Manner

**DOI:** 10.1155/2018/3424956

**Published:** 2018-07-12

**Authors:** Minghai Shao, Tienan Bi, Wenxiu Ding, Changhui Yu, Caiping Jiang, Haihua Yang, Xinchen Sun, Min Yang

**Affiliations:** ^1^Department of Radiation Oncology, The First Affiliated Hospital of Nanjing Medical University, Nanjing, Jiangsu Province 210029, China; ^2^Department of Radiation Oncology, Taizhou Hospital, Wenzhou Medical University, Linhai, Zhejiang Province 317000, China; ^3^Department of Gastrointestinal Surgery, Taizhou Hospital, Wenzhou Medical University, Linhai, Zhejiang Province 317000, China; ^4^Jiangsu Institute of Nuclear Medicine, Wuxi, Jiangsu Province 214063, China

## Abstract

Radiotherapy is an important strategy for rectal cancer patient treatment. However, the efficiency of radiation is usually poor, especially in patients with advanced stage rectal cancer due to the radio-resistance developed. At the present study, OCT4 was found to play a critical role in radio-resistance development in human rectal cancer cells by improving the epithelial-mesenchymal transition process (EMT). Endogenous OCT4 expression could confer resistant phonotype on human rectal cancer cells, which was supported by the data from clonogenic forming assay and cell cycle arrest recovering experiment. EMT related transcription factor ZEB1 might take part in the radio-resistance induced by OCT4, as its expression could be upregulated by OCT4 and its silence could reverse the OCT4 induced resistance to radiation in SW480 cells. More interestingly, CHK1 was also upregulated in OCT4/ZEB1 dependent manner conferring stronger DNA damage repair activity on cancer cells, which might explain the underlying mechanisms why OCT4/ZEB1 axis could promote the resistance of human rectal cancer cell to radiation. Taken together, our results provided a novel mechanism for radio-resistance development in human rectal cancer cells and a new target to overcome this resistance.

## 1. Introduction

Rectal cancer, as a disease in which malignant cells form in the tissue of the rectum, is the fifth most frequently diagnosed cancer. In 2017, an estimated 39,910 new cases of rectal cancer occurred in the United States [1]. Individual or combined applications of surgery, radiation therapy, chemotherapy, and targeted therapy are the major strategies for rectal cancer treatment. Particularly, the neoadjuvant chemoradiation is routinely used on the patients with stage II to III rectal cancers [2]. However, the 5-year overall survival rate of rectal cancer patients in advanced stage is still markedly low due to the limited therapy efficiency [3]. One of reasons resulting in the poor survival was the resistance developed during the treatments towards to drug and radiation.

As numerous previous studies reported, radiation causes cell death by inducing single- or double-strands DNA breaks in tumor cells which are under actively dividing [4]. And the major reasons for radiation therapy failure are the intrinsic or acquired radio-resistance developed by cancer cells with increased DNA damage repair activity [5]. In response to DNA damage, two sensors, the RAD9–HUS1–RAD1 (9–1–1) complex and the MRE11–RAD50–NBS1 (MRN) complex, are recruited to the DNA damage sites to induce the cell cycle arrest, which facilitate the recruitment of phosphorylated histone H2AX (*γ*H2AX) surrounding the DNA breaks to initiate the DNA damage repair [6]. Increasing number of studies indicated that cancer cells could develop radio-resistance by abnormally activating the DNA damage response (DDR) [7].

Recently, cancer stem cells were found in promoting the radio-resistance of tumor by enhancing the DDR. Moreover, the resistance capacity of the tumor is highly associated with the level of cancer stem cell [8]. As the key transcriptional factor for embryonic stem cells, OCT4 is essential for stem cell self-renewal and cell differentiation [9]. Furthermore, OCT4 also plays an important role in tumorigenesis, therapy resistance, and prognosis [10]. As to cancer stem cells, the expression level of OCT4 contributes to maintain their stemness that is critical for developing resistance to chemotherapeutics or radiation [10]. Several studies reported that OCT4 is closely associated with the DNA damage response of cancer cells towards to irradiation [11, 12]. High expression level of OCT4 in tumor cells indicates resistance to radiotherapy and was an important predictor for the poor survival of the patients with cervical squamous cell carcinomas [11]. OCT4 and its target gene,* CIP2A*, were both linked to increased aggressiveness and radio-resistance in human head and neck squamous cell carcinoma cells [12]. Interestingly, the abundance of OCT4 can be used to predict the cancer distant recurrence and poor prognosis after preoperative chemoradiation treatment in rectal cancer patients [13]. However, the detailed mechanisms underlying the role of OCT4 played in radiation resistance developed in cancer cells, including rectal cancer, remained to be further elucidated.

The epithelial-mesenchymal transition (EMT) is a process by which epithelial cells lose their cell polarity and cell-cell adhesion and become mesenchymal stem cells. EMT is essential for numerous developmental processes, including mesoderm formation and neural tube formation, as well as being involved in wound healing, organ fibrosis, and initiation of cancer cell metastasis [14]. Moreover, the EMT transdifferentiation program can generate cells with stem-like properties [14]. Therefore, as to cancer cells, EMT not only is the initial step for cell migration, but also plays a critical role in regulating chemo- and radiotherapy resistance [15]. Various transcription factors, including Twist, Snail, Slug, and ZEB1, can induce EMT. However, little is known whether EMT itself or other specific regulators cause cancer stem-like properties. Interestingly, OCT4 was shown to have a regulatory role in EMT process of cancer cells [16]. In human liver cancer, OCT4 could active LEF1/*β*-catenin dependent WNT signaling pathway and promote epithelial-mesenchymal transition [16]. These findings suggest that OCT4 might possess an effect on tumor responses for chemo- or radio-treatment via EMT pathway.

To date, little is known about the role of OCT4/EMT axis in human rectal cancer cells. Therefore, whether OCT4 is associated with radio-resistance in human rectal cancer was determined and the underlying mechanism was also elucidated in the present study. Our data indicated that high level of OCT4 was positively accompanied with radiation resistance of human rectal cancer cell lines. Ectopic expression of OCT4 in SW480 cells confers the cells much higher resistance to irradiation therapy with stronger DNA damage repair ability and more active cell migration. More interestingly, OCT4 could upregulate ZEB1 expression and subsequently promote the EMT process of SW480 cells. Knockdown of ZEB1 can effectively reverse the radiation resistance of SW480 cells induced by OCT4 overexpression. In conclusion, our results indicated that OCT4 can confer radiation resistance and migration activity of human rectal cancer cells by enhancing EMT in ZEB1 dependent manner for the first time.

## 2. Material and Methods

### 2.1. Cell Culture and Antibodies

Human rectal carcinoma cell lines HT29 and SW480 were obtained from American Type Culture Collection. Both cell lines were cultured in 37°C humidified 10% CO_2_ incubator with DMEM medium (Invitrogen, Inc., Carlsbad, CA) including 10% (v/v) fetal bovine serum (FBS) and 2 mM L-glutamine.

Antibodies against GAPDH, OCT4, ZEB1, *γ*-H2AX, and CHK1 were purchased from Cell Signaling Technology (California, USA).

### 2.2. Plasmids and Transfection

The full-length human* OCT4* coding sequence fragment (CCDS34391.1) was synthesized and subcloned into pcDNA3.1 vector to construct OCT4 overexpression plasmid, which was verified by sequencing. After cells were seeded for overnight, 2 *μ*g OCT4 overexpression plasmid or empty vector as control was transfected using Lipofectamine 3000 (Invitrogen) according to the manufacturer's instructions. Western blotting was used to validate the expression efficiency of the plasmid after cells were transfected for 24h and 48h.

siRNA against ZEB1 (sense sequence: 5'-CCAAUAAGCAAACGAUUCUGA-3') and scramble siRNA (sense sequence: 5'-UUCUCCGAACGUGUCACGU-3') as negative control were obtained from Genepharma (Shanghai, China). 1.2 *μ*g of ZBE1 siRNA or negative siRNA was subjected to cells using Lipofectamine 3000 according to the manufacturer's instructions and the silence efficiency was determined by western blotting assay after 24h incubation.

### 2.3. Clonogenic Survival Assay

Exponentially growing rectal cancer cells cultured in 100 mm dish were irradiated at 0.61 Gy/min per exposure by X-ray irradiator (RS 2000 Biological System irradiator, Rad Source, USA). After irradiation, the cells were harvested for clonogenic survival analysis. Survival after radiation exposure was defined as the ability of the cells to maintain their clonogenic capacity and to form colonies. Briefly, cells were trypsinized, counted, and seeded 500 cells per well into 6-well plate. After incubation for 15 days, colonies were stained with 0.5% crystal violet solution for 30 minutes and counted manually. Experiments were performed at least 3 times independently.

### 2.4. Western Blotting Analysis

The western blotting assay was performed as published previously [17]. After treatment indicated, the cells were lysed with total lysis buffer (2.1 *μ*g/ml aprotinin, 0.5 *μ*g/ml leupeptin, 4.9 mM MgCl2, 1 mM orthovanadate, 1% Triton X 100, and 1 mM PMSF). Protein concentrations were determined by BCA kit (Thermo Pierce). 20 *μ*g proteins were resolved in denaturing SDS-PAGE gel and transferred to PVDF membrane (Millipore, Billerica, MA). After blocking with 5% fat-free dry milk, the membranes were washed by PBST (PBS containing 0.1% Tween 20) and incubated with primary antibodies followed by respective horseradish peroxidase conjugated secondary antibodies. Signals were visualized with enhanced ECL chemiluminescence detection reagents (Thermo Pierce) and visualized on X-ray film (Fuji Photo Film, Tokyo, Japan). GAPDH was used as reference to determine the protein relative level.

### 2.5. Real-Time PCR

Total RNA was isolated using the Trizol (Invitrogen, Carlsbad, MA) according to the manufacturer's protocols. Complementary DNA synthesis was performed using the PrimeScript RT Reagent Kit (TaKaRa, Osaka, Japan). The expression levels of* OCT4* mRNA (forward: 5'- CCCGAAAGAGAAAGCGAACC -3'; reverse 5'- CCCCTG AGAAAGGAGACCCA -3') and* ZEB1* mRNA (forward: 5'- ACACGACCACAGA TACGGCA -3'; reverse 5'- ATGGGAGACACCAAACCAAC -3') were evaluated using SYBR green PCR master mix (Applied Biosystems) and normalized to *β*-actin (forward: 5'-AGCACAGAGCCTCGCCTTTGC-3'; reverse 5'-CTGTAGCCGCG CTCGGTGA G-3'). Real-Time PCR amplification was performed in ABI 7500 Fast Real-Time PCR system (Applied Bioscience, Foster City, CA) according to manufacturer's procedure for relative quantification. All of the reactions were performed in triplicate.

### 2.6. FACS Assay

After treatment indicated, cells were harvested and washed with cold PBS twice and fixed with cold 70% ethanol at least overnight. Then, cells were centrifuged and washed with cold PBS twice again and resuspended the pellet in PBS and stained cells with PI solution in concentration of 10 *μ*g/ml for 30 min. Cells were then analyzed with flow cytometry (FACS Calibur, BD Biosciences).

### 2.7. In Vitro Migration Assays

For the cell migration assay, the transwell (8 *μ*m pore size with poly-carbonate membrane, Corning) was used according to the manufacturer's protocols. The cells were seeded into the upper chambers and cultured in serum-free DMEM medium after irradiation to block their proliferation. The lower compartment was filled with DMEM medium with 10% FBS. After 12h incubation, the cells remaining in the upper chamber were removed, and the cells at the bottom of the insert were fixed, stained in 0.5% crystal violet solution for 30 minutes, and counted under a microscope (Olympus Corp., Tokyo, Japan). The results were averaged overthree independent experiments.

### 2.8. Chromatin Immunoprecipitation (CHIP) Assay

ChIP assay was performed using the Chromatin Immunoprecipitation Assay kit according to the manufacturer's instruction (Cell Signaling Technology). Briefly, cell cultures in 15 mm dish were fixed to cross-link histones to DNA. Cells were washed and detached from the dish by scraping followed by addition of lysis buffer. After 10 min incubation on ice, the lysis product was treated with Micrococcal Nuclease 10011 to digest DNA to length of approximately 150-900 bp. After centrifugation at 14,000 rpm for 10 min at 4°C, the sonicated cell supernatants were diluted with Dilution Buffer and aliquots of samples were saved as the input DNA for quantization of the amount of total DNA. For immunoprecipitation, 1 *μ*g antibody against OCT4 or normal IgG as negative control was added to the supernatants and incubated overnight at 4°C with rotation. Immunocomplexes were collected using Protein G Agarose beads for 1 h at 4°C. Following the wash, the immunocomplexes were recovered by resuspending in elution buffer at room temperature for 15 min. DNA-protein complexes as well as the input DNA were reverse cross-linked at 65°C for 4 h and treated with proteinase K at 45°C for 1 h. DNA was purified and was subjected to PCR with primers: forward, 5'- TGGAAGGGAAGGGAAGGGAG -3' and reverse, 5'- TTGAGGGGCGAGGGAAAAGT -3'. Amplification was carried out for 35 cycles with denaturation at 94°C for 30 s, annealing at 58°C for 30 s, and extension at 70°C for 40 s. PCR products were analyzed on a 1.5% agarose gel.

### 2.9. Statistical Analysis

In all experiments, data were expressed as mean ± standard deviation (SD). A significant difference of the sample's value from that of the respective controls in each experiment condition was assessed using Student's unpaired *t*-test with *p* value < 0.05 being regarded as statistically significant.

## 3. Results

### 3.1. OCT4 Is Positively Associated with the Irradiation Resistance of Human Rectal Cancer Cell

**Figure 1:**
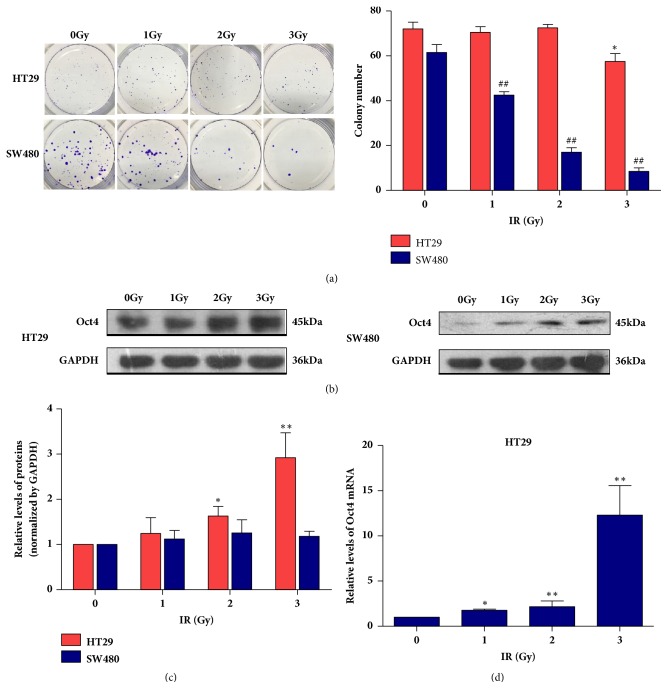
OCT4 were positively associated with radio-resistance of human rectal cancer cells. (a) HT29 and SW480 cells were exposed to irradiation with indicated dose followed by another 24 hours incubation, and then cells were harvested and seeded 500 cells/well into six-well plate for 15-day incubation for clonogenic survival assay. Data are presented as mean ± SD, *n* = 3. ^*∗*/#^*P* < 0.05 versus control; ^*∗∗*/##^*P* < 0.01 versus control. (b) and (c) OCT4 protein expression and its variation during irradiation were detected by western blotting assay. Data are presented as mean ± SD, *n* = 3. ^*∗*^*P* < 0.05 versus control; ^*∗∗*^*P* < 0.01 versus control. (d)* OCT4* mRNA expression and its variation during irradiation were detected by Real-Time PCR. Data are presented as mean ± SD, *n* = 3. ^*∗*^*P* < 0.05 versus control; ^*∗∗*^*P* < 0.01 versus control.

At the present study, we applied human rectal cancer cell lines HT29 and SW480 to determine their sensitivity to irradiation. After exposure to 0, 1, 2, or 3Gy dose of radiation followed by 24h incubation, cells were harvested to perform clonogenic survival assay. Our results indicated that HT29 cells presented higher resistance to radiation compared to SW480 cells (Figure 1(a)), which was consistent with previous publication [18]. The OCT4 expression profiling in these two cell lines under different doses of radiation was also detected by western blotting assay. As expected, the basal expression of OCT4 was significantly higher in HT29 cells than SW480 cells (Figure 1(b)), which also is supported by the mRNA levels (data not shown). More interestingly, the OCT4 levels were upregulated in both two cell lines in a dose dependent manner responding to irradiation treatment. And the increase was much higher in HT29 cells (Figures 1(b) and 1(c)).

**Figure 2:**
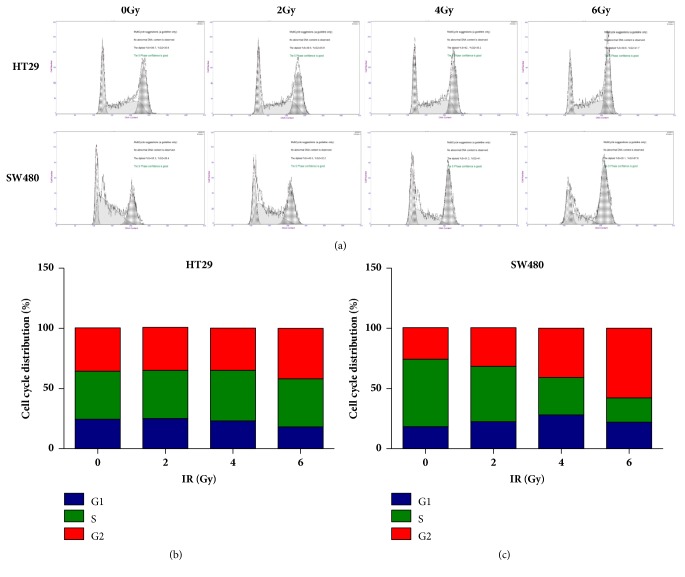
Radiation induced cell cycle arrest in SW480 cells. (a) Both HT29 and SW480 cells were treated with indicated dose of radiation, and cells were harvested after 24-hour incubation. Cell cycle distributions of both cell lines were determined by PI staining followed by FACS assay. (b) HT29 and (c) SW480 cell. Representative results from three independent experiments.

Furthermore, the level of* OCT4 mRNA* in HT29 cell after radiation was measured using Real-Time PCR experiment. As shown in Figure 1(d),* OCT4* expression also increased at mRNA level in HT29 cells under irradiation in a dose dependent manner. Besides, there was weak upregulation of* OCT4* mRNA in SW480 cells as well (data not shown). Finally, cell cycle distributions of these two cell lines under different doses of irradiation were determined by FACS assay to evaluate DNA content using PI staining. As shown in Figure 2, significant cell cycle arrest was observed in SW480 cells treated with 4Gy dose of radiation. But there was no significant cell cycle arrest in HT29 cells even under 6Gy dose of radiation.

Taken together, our data implied that OCT4 might take part in the development of radiation resistance in cancer cells.

### 3.2. Ectopic Expression of OCT4 Confers Resistance to Radiation on SW480 Cells

**Figure 3:**
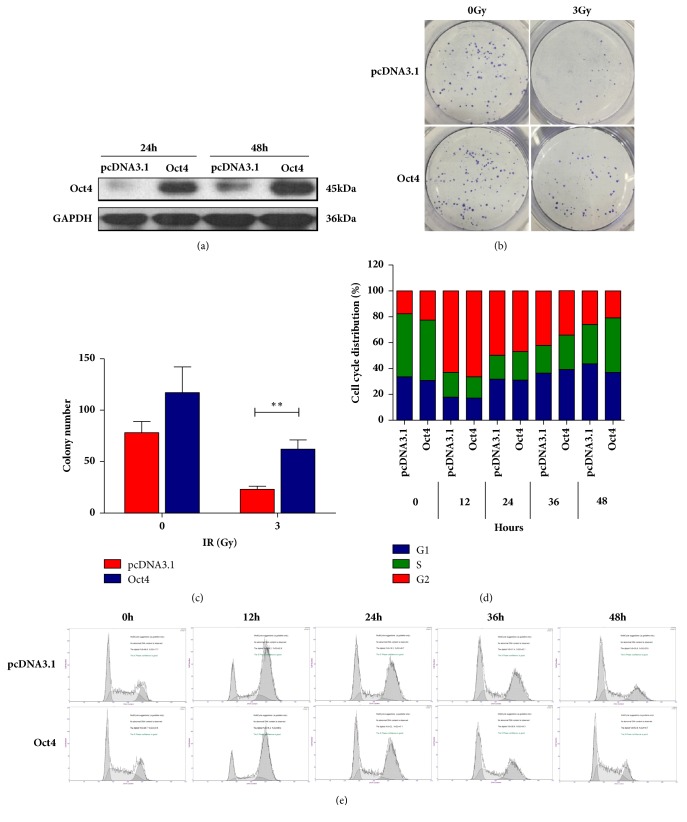
OCT4 conferred SW480 cells radio-resistance. (a) SW480 cells were transfected with OCT4 overexpression plasmid. The expression efficiency was validated by western blotting in indicated time. (b) and (c) After being transfected with OCT4 overexpression plasmid for 24 hours, SW480 cells were exposed to 3Gy irradiation followed by another 24-hour incubation and clonogenic survival assay was performed. Data are presented as mean ± SD, *n* = 3. ^*∗∗*^*P* < 0.01 versus control. (e) SW480 cells were transfected with OCT4 overexpression plasmid and the cell cycle distribution was determined by PI staining followed FACS assay in indicated time after 3Gy radiation treatment. Representative results from three independent experiments.

To further confirm the roles of OCT4 in irradiation resistance regulation of human rectal cancer cells, we constructed OCT4 overexpression plasmid and subjected it to SW480 cells. The expressing efficiency of OCT4 overexpression plasmid in SW480 cells was validated by western blotting assay after transfection for 24h and 48h. Our data indicated that significant elevated level of exogenous OCT4 was observed in SW480 cells after being transfected with OCT4 overexpression plasmid for 24h and increased more at 48h (Figure 3(a)). In order to determine whether OCT4 ectopic expression could potentiate the resistance to radiation in SW480 cells, we performed clonogenic forming assay and FACS assay, respectively, to evaluate the proliferation activity and cell cycle progress.

The result of clonogenic forming assay also revealed that ectopic expression of OCT4 effectively increased SW480 cells' tolerance to irradiation (Figures 3(b) and 3(c)). Furthermore, we applied FACS assay to measure cell cycle and found SW480 cells with endogenous OCT4 possessed much stronger activity in recovering cell cycle arrest compared to ones transfected with the empty vector (Figures 3(d) and 3(e)). The cells with overexpressed OCT4 presented weak cell cycle arrest compared with the control, although the cells in both two groups underwent serious cell cycle arrest in 12h after radiation and subsequently recovered (Figure 3(d)). However, the data also indicated that cells with increased OCT4 expression show a much quicker recovering and almost withdrew from cell cycle arrest in 48h after radiation (Figure 3(d)).

All the data present supported that OCT4 indeed played an important role in regulating radiation resistance in human rectal cancer cells.

### 3.3. OCT4 Promotes EMT Process of SW480 Cells by Upregulating ZEB1 with Migration Activity Enhanced

**Figure 4:**
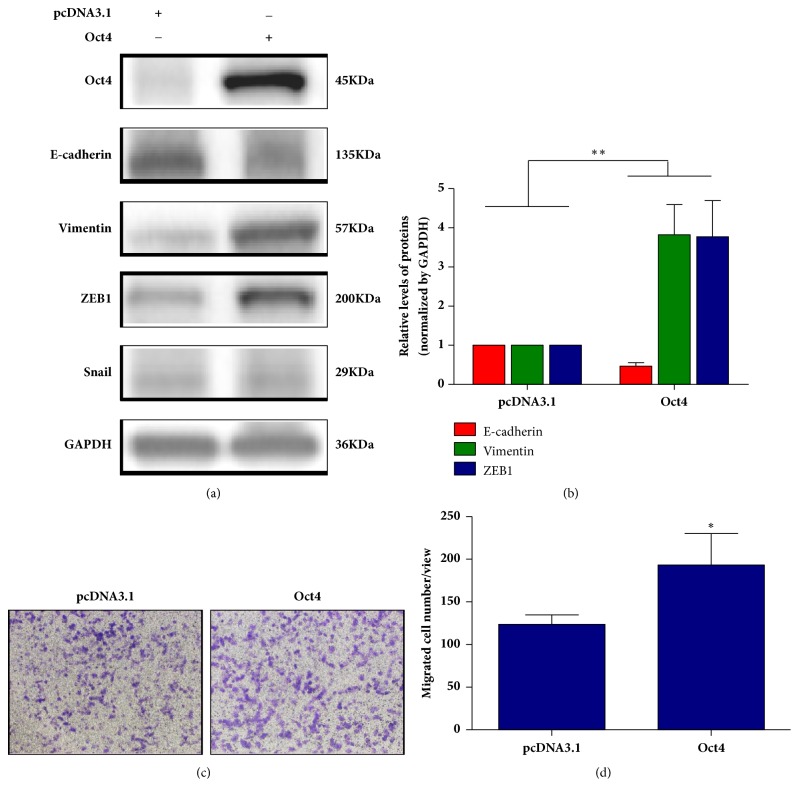
OCT4 promoted EMT and migration activity in SW480 cells. (a) and (b) SW480 cells were transfected with OCT4 overexpression plasmid for 24h, and the expression of proteins indicated was validated by western blotting assay. (c) and (d) Transwell experiment was used to compare the migration activities of SW480 cells transfected with OCT4 overexpression plasmid or empty vector. Cells were preirradiated to stop proliferation before this assay. Data are presented as mean ± SD, *n* = 3. ^*∗*^*P* < 0.05 versus control; ^*∗∗*^*P* < 0.01 versus control.

The fact that EMT plays a critical role in regulating chemo- and radiotherapy resistance prompted us to investigate whether increased OCT4 could promote EMT in SW480 cells. We detected the protein level of main EMT related transcriptional factors, including ZEB1 and Snail, as well as key EMT marker proteins, such as E-cadherin and vimentin in SW480 cell with OCT4 overexpressed. Interestingly, we observed that overexpression of OCT4 significantly increased the expression of ZEB1 as well as N-cadherin but suppressed E-cadherin (Figures 4(a) and 4(b)). However, only slight upregulation was observed in Snail expression when endogenous OCT4 was involved (Figures 4(a) and 4(b)).

To further conform whether OCT4 overexpression could promote SW480 cell migration along with the induction of EMT, transwell assay was performed. As expected, OCT4 could indeed significantly promote the migration ability of SW480 cells (Figures 4(c) and 4(d)). All these data suggested that OCT4 could induce EMT in rectal cancer cell SW480 and subsequently active the cell migration.

### 3.4. ZEB1 Is Indispensable for OCT4 Induced Irradiation Resistance in Human Rectal Cancer Cell

**Figure 5:**
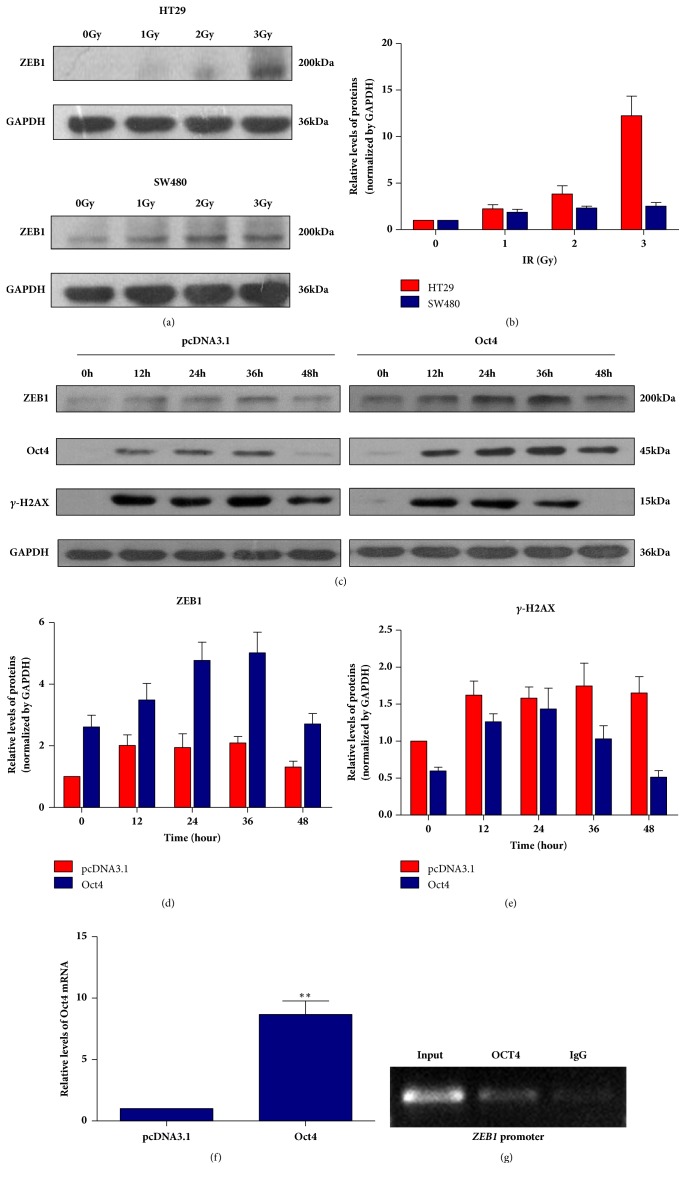
ZEB1 was involved in OCT4 induced radio-resistance in human rectal cancer cells. (a) and (b) HT29 and SW480 cells were treated with indicated dose of radiation. ZEB1 protein expression and its variation during irradiation were detected by western blotting. (c) SW480 cells were transfected with OCT4 overexpression plasmid and the indicated proteins expression ((d) ZEB; (e) *γ*-HA2X) was determined in indicated times after 3Gy radiation treatment. (f) SW480 cells were transfected with OCT4 overexpression plasmid or the control for 24h. ZEB1 mRNA levels were detected by Real-Time PCR. Data are presented as mean ± SD, *n* = 3. ^*∗∗*^*P* < 0.05 versus control. (g) CHIP assay was performed using OCT4 antibody and corresponding IgG in SW480 cells after OCT4 overexpression plasmid transfection. Representative results from three independent experiments.

Although ZEB1 was upregulated by OCT4, the exact roles of ZEB1 in radiation resistance of human rectal cancer remained unknown. We performed western blotting assay to measure the ZEB1 expressions in SW480 cells after treating with 0, 1, 2, or 3Gy dose of radiation. As shown in Figures 5(a) and 5(b), basal ZEB1 of SW480 seemed higher compared with HT29 cells, while ZEB1 upregulated responding to radiation treatment in both cell lines in a dose dependent manner like OCT4. However, HT29 cells expressed much higher ZEB1 under radiation treatment compared with SW480 cells, which was also consistent with OCT4 expression profiling induced by radiation (Figure 1(a)).

Furthermore, we measured the ZEB1 expression change at different time points after radiation treatment in SW480 cells overexpressed OCT4. As shown in Figures 5(c) and 5(d), OCT4 and ZEB1 were significantly increased side by side in SW480 cells transfected with OCT4 overexpression plasmid when exposed to radiation. More importantly, *γ*-HA2X, the marker for DNA damage repair persisted much longer time in control cells (Figures 5(c) and 5(e)). These data indicated that OCT4/ZEB1 axis might be in need for DNA damage repair induced by radiation.

We also measured the* ZEB1* mRNA expression in SW480 cells with OCT4 overexpressed. The date from Real-Time PCR revealed that the ectopic expression of OCT4 significantly increased ZEB1 mRNA level (Figure 5(f)) which suggested OCT4 could activate the* ZEB1* gene transcription. Therefore, we analyzed the ZEB1 promoter sequence using an online transcription factor binding predict software TFBIND and found that a dozen of potential binding sites of OCT4 located in this promoter. CHIP was subsequently performed and the result indicated that OCT4 indeed binds to* ZEB1* gene promoter (Figure 5(g)).

**Figure 6:**
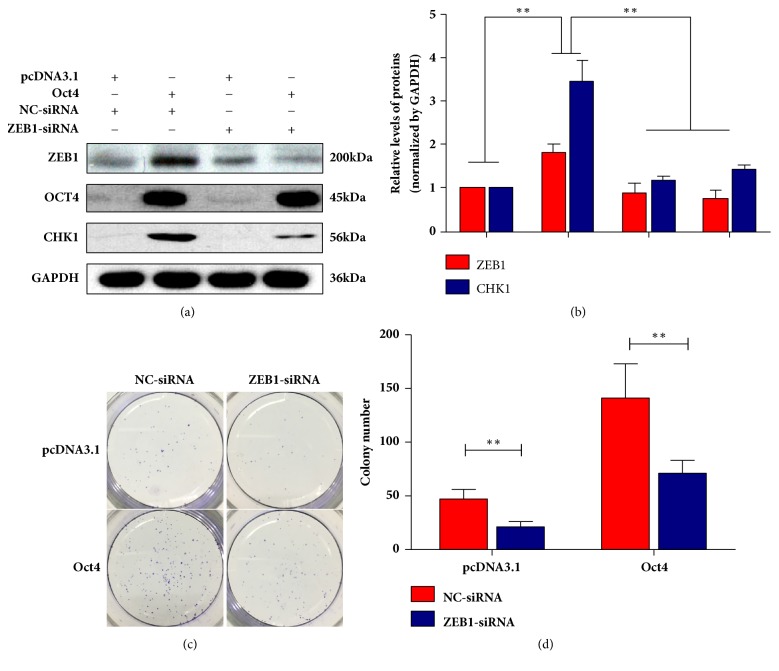
ZEB1 might contribute to OCT4 induced radio-resistance in human rectal cancer cells through upregulating CHK1 expression. (a) and (b) SW480 cells were transfected with OCT4 overexpression plasmid and siRNA-ZEB1 alone or combined for 24h. The indicated protein expressions were detected by western blotting. (c) SW480 cells were transfected with OCT4 overexpression plasmid and siRNA-ZEB1 alone or combined for 24h. After 3Gy radiation exposure followed by another 24 h incubation, clonogenic survival assay was performed. Data are presented as mean ± SD, *n* = 3. ^*∗∗*^*P* < 0.01 versus control.

Finally, inhibition ZEB1 by ZEB1 siRNA was used to validate whether OCT4 induced irradiation resistance is dependent on ZEB1. SW480 cells were cotransfected with OCT4 overexpression plasmid and ZEB1 siRNA. The expression profiling of OCT4 and ZEB1 were determined by western blotting assay (Figure 6(a)). The results of clonogenic survival assay showed radiation resistance of SW480 cells induced by OCT4 overexpressed elapsed for ZEB1 effective silence (Figures 6(c) and 6(d)). Besides, CHK1 was upregulated by ectopic expression of OCT4 and also impaired by ZEB1 silence (Figures 6(a) and 6(b)). All these findings suggested that OCT4 could increase the irradiation resistance in a ZEB1 dependent manner that probably was with CHK1 involved.

## 4. Discussion

For patients with late-staged rectal cancer, individual or combined applications of chemotherapy and irradiation are first line therapeutic strategies [19]. Although the tumors developed from the bowel were usually sensitive to irradiation, the treatment benefit was unfortunately limited due to intrinsic or acquired radio-resistance of cancer cells [20]. Overcoming this kind of resistance could improve the prognosis of rectal cancer patients. Therefore, it is very critical to understand the potential mechanisms of tumorous radio-resistance, to help us find the targets by which sensitivity of the cancer cells could be recovered. Besides, it is also important to establish a molecular biomarker which can predict the response of tumor to irradiation prior to treatment.

The molecular basis for irradiation resistance of tumor is really complicated, as almost any abnormal regulations in DNA damage sensing and signaling, cell cycle check point, DNA damage repair, and cell apoptosis can confer tumor cell resistance to radiation [20]. Recent study revealed that WNT/*β*-catenin signaling pathway contributed to chemoradiation resistance of colorectal cancer, inhibition of *β*-catenin by siRNA, or small molecular inhibitor could effectively reverse this resistance [21]. In human colorectal cancer, fibroblast growth factor receptor 4 (FGFR4) is usually upregulated as an oncogene. Silence of FGFR4 in radio-resistant HT29 decreased the cell survival after irradiation, while its overexpression in radio-sensitive SW480 conferred the stronger DNA damage repair ability on the tumor cells [22]. In a preclinical rectal cancer model, inhibition of exportin 1, a mediator for the nuclear export of critical proteins required for rectal cancer proliferation and treatment resistance, resulted in an increased apoptosis and decreased proliferation under single irradiation [23]. Furthermore, extra high expression levels of some regulators of apoptosis such as XIAP were also positively related to tumorous radio-resistance [24].

As mentioned previously, the EMT process is not only the initial step for metastasis of cancer cell, but also critical for their treatment resistance. The EMT process confers several stem cell-like properties on the cancer cells which might account for the resistant responses of cancer cells to chemotherapy or irradiation [15, 25]. On the other hand, some stem cell related factors like OCT4 and Nanog could promote the EMT process in human colorectal cancer cells, and OCT4 was also found to be associated with rectal cancer distant recurrent after chemoradiation treatment [13, 16, 26]. All these findings suggested that OCT4 could be a molecular biomarker for response predictor of cancer to irradiation and a potential target by which we can overcome this resistance. However, this is no publication about whether OCT4 possessed a direct regulatory role in radio-resistance of human rectal cancer cells.

At the present study, we found OCT4 was highly expressed in radio-resistant HT29 cells, while the level is very low in radio-sensitive SW480 cells, indicating OCT4 could also act as a molecular predictor for radiation response in human rectal cancer. Furthermore, endogenous expression of OCT4 could generate the radio-resistance phenotype in SW480 cells accompanying with stronger DNA damage repair activity. Next, we examined whether OCT4 overexpression could influence the EMT level of human rectal cancer cells. As respected, ectopic expression of OCT4 increased the expression of EMT related adherent protein such that vimentin with E-cadherin decreased. OCT4 expression also upregulated the EMT related transcriptional factor ZEB1 and inhibition of ZEB1 with siRNA could effectively attenuate the radio-resistance induced by OCT4. More importantly, we found that OCT4 could promote the mRNA expression of* ZEB1*, which suggested OCT4 might activate the* ZEB1* transcription. We analyzed the promoter sequence of* ZEB1* gene with online transcriptional factor biding site predict software and found that there are a dozen OCT4 potential binding sites located in the promoter, which was also supported by the results of CHIP experiment.

Although the detailed mechanisms for how OCT4/ZEB1 pathway regulated the radio-resistance of rectal cancer cells remained to be further documented, it looks like that the regulation of DNA damage repair might be involved, as ZEB1 was found to could stabilize CHK1 in response to DNA damage in human breast cancer [27]. But, whether and how ZEB1 could influence the components of DNA damage repair machine in rectal cancer cells remain to be further confirmed.

## 5. Conclusions

Taken together, our study revealed that OCT4 conferred radio-resistance in human rectal cancer cells by promoting EMT process in a ZEB1 dependent manner for the first time. In future, more experiments will be performed to further elucidate the underlying mechanism to validate OCT4 as a response predictor and therapeutic target for radiation in human rectal cancer cells.

## Data Availability

The data used to support the findings of this study are included within the article.
